# Case of seasonal reassortant A(H1N2) influenza virus infection, the Netherlands, March 2018

**DOI:** 10.2807/1560-7917.ES.2018.23.15.18-00160

**Published:** 2018-04-12

**Authors:** Adam Meijer, Corien M Swaan, Martin Voerknecht, Edin Jusic, Sharon van den Brink, Lisa A Wijsman, Bettie CG Voordouw, Gé A Donker, Jacqueline Sleven, Wendelien W Dorigo-Zetsma, Sanela Svraka, Michiel van Boven, Manon R Haverkate, Aura Timen, Jaap T van Dissel, Marion PG Koopmans, Theo M Bestebroer, Ron AM Fouchier

**Affiliations:** 1 Centre for Infectious Disease Control, National Institute for Public Health and the Environment, Bilthoven, the Netherlands; 2 General practitioner participating in the Primary Care Database sentinel surveillance coordinated by NIVEL Netherlands institute for health services research, Utrecht, the Netherlands; 3 Coordinator NIVEL Primary Care Database sentinel surveillance, NIVEL Netherlands institute for health services research, Utrecht, the Netherlands; 4 Municipal Health Services ‘Gooi en Vechtstreek’, Bussum, the Netherlands; 5 Central Bacteriology and Serology Laboratory, Tergooi Hospitals, Hilversum, the Netherlands; 6 Department Viroscience, Erasmus University Medical Center, Rotterdam, the Netherlands

**Keywords:** human influenza virus, reassortant, influenza-like illness - ILI, surveillance, epidemiology, emerging or re-emerging diseases

## Abstract

A seasonal reassortant A(H1N2) influenza virus harbouring genome segments from seasonal influenza viruses A(H1N1)pdm09 (HA and NS) and A(H3N2) (PB2, PB1, PA, NP, NA and M) was identified in March 2018 in a 19-months-old patient with influenza-like illness (ILI) who presented to a general practitioner participating in the routine sentinel surveillance of ILI in the Netherlands. The patient recovered fully. Further epidemiological and virological investigation did not reveal additional cases.

In the routine sentinel general practitioner (GP) surveillance of influenza in the Netherlands, a new seasonal reassortant A(H1N2) influenza virus was identified in natural human infection in March 2018. Here we report the case detection, molecular characterisation of the virus and the results of follow-up epidemiological and virological investigation.

## Influenza surveillance in the Netherlands

In the Netherlands, influenza surveillance is a collaborative effort of the National Influenza Centre (NIC) (consisting of the National Institute for Public Health and the Environment (RIVM) in Bilthoven and the Erasmus University Medical Centre (Erasmus MC) in Rotterdam) and the NIVEL Primary Care Database sentinel surveillance in Utrecht [1,2]. The GPs participating in the sentinel surveillance report weekly the incidence of influenza-like illness (ILI) according to the case definition by Pel (sudden onset of symptoms, fever of at least 38 °C and at least one of the following: cough, rhinorrhoea, sore throat, frontal headache, retrosternal pain, myalgia) [3]. They take nose and throat swabs for influenza virus detection by real-time RT-PCR at the NIC from a systematic selection of patients presenting with ILI or another acute respiratory infection (ARI). Diagnostic GP and hospital laboratories forward influenza virus-positive specimens for further characterisation to the NIC. All influenza viruses are subtyped by real-time RT-PCR and a subset is further characterised by sequencing, virus isolation and haemagglutination inhibition and neuraminidase inhibition assays.

## Case detection

**Figure 1:**
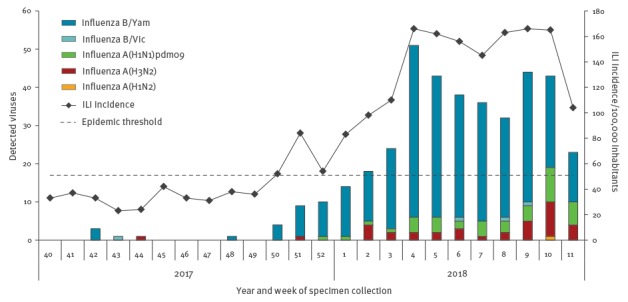
ILI incidence and number of specimens from patients with ILI collected by sentinel general practitioners in which influenza virus was detected, the Netherlands, 21 March 2018 (n = 395)

In the Netherlands, the 2017/18 influenza epidemic has been dominated by influenza viruses of the B/Yamagata lineage (Figure 1). Since the beginning of March, both A(H3N2) and A(H1N1)pdm09 influenza viruses have been increasingly detected. By week 11/2018 the incidence of ILI had been above the epidemic threshold for 14 weeks. In week 10 on 5 March, a sentinel GP was consulted for a 19 months-old patient with acute onset of ILI on 2 March characterised by fever, malaise, sore throat, coughing, shortness of breath, rhinorrhoea and diarrhoea.

ILI: influenza-like illness.

Left axis: influenza virus-positive specimens from patients with ILI; right axis: ILI incidence per 100,000 inhabitants. Data are presented by week of specimen collection (ISO week numbering).

Nose and throat swabs were collected during the consultation with permission of the accompanying parent and combined in one tube with virus transport medium submitted to the NIC. The specimen collection form indicated that the patient had not travelled abroad, was not vaccinated against influenza, did not use influenza antiviral drugs and did not have underlying disease. In the routine procedure, the specimen tested positive for influenza virus type A on 8 March using RT-PCR targeting the M segment. It tested positive for subtypes H1pdm09 and N2 and negative for subtypes H3 and N1pdm09 on March 13 using H and N subtype-specific RT-PCRs. Sanger sequencing showed on 16 March that the haemagglutinin (HA) gene was derived from A(H1N1)pdm09 and the neuraminidase (NA) gene from A(H3N2) human seasonal influenza viruses. That the influenza A(H1N2) virus was detected and subtyped with RT-PCR assays routinely used in the surveillance indicates that widely used RT-PCR assays for human seasonal influenza viruses are appropriate to detect the reassortant virus and to identify its H and N subtype.

## Virus characterisation

MinION nanopore whole genome sequencing confirmed on 18 March that the patient was infected by a seasonal reassortant A(H1N2) influenza virus, with the HA and non-structural (NS) segments derived from seasonal A(H1N1)pdm09 influenza virus and all other segments (polymerase components PB2, PB1 and PA, nucleoprotein (NP), NA and matrix (M)) from seasonal influenza A(H3N2) virus. The genome sequence of influenza virus A/Netherlands/10407/2018 H1N2 is available from the GISAID EpiFlu database (EPI_ISL_304183). 

**Figure 2:**
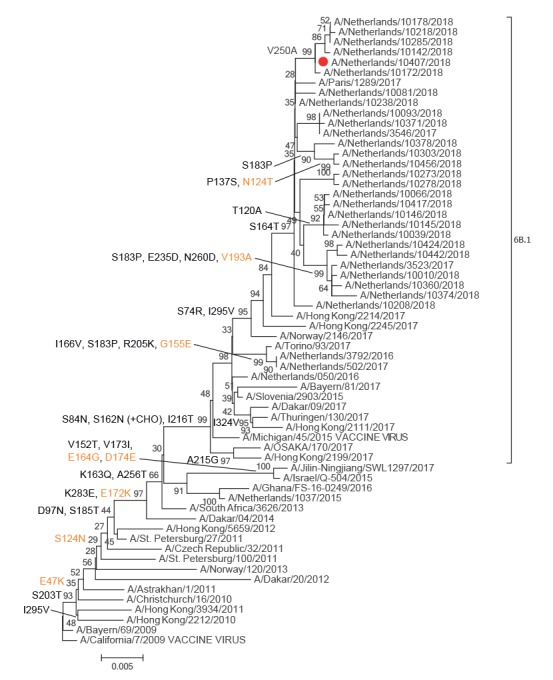
Phylogenetic analysis of the influenza A(H1N2) virus haemagglutinin genome segment in the context of a reference influenza virus data set^a^ and recent influenza viruses from the Netherlands

**Figure 3:**
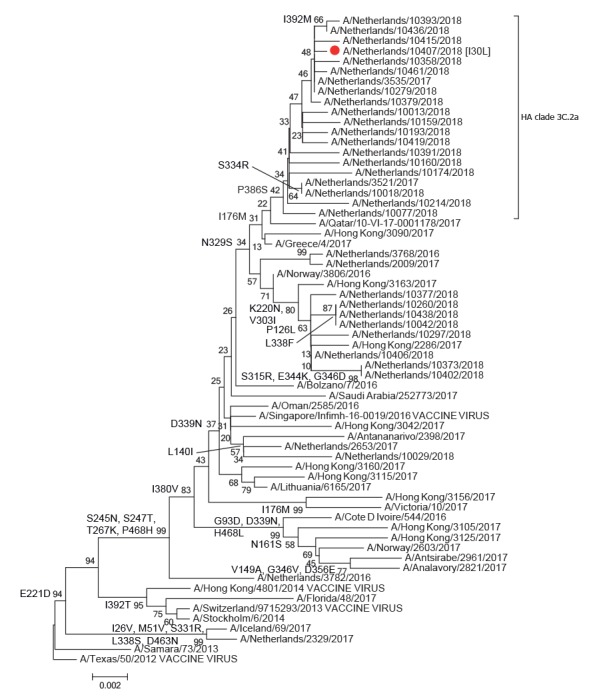
Phylogenetic analysis of the influenza A(H1N2) virus neuraminidase genome segment in the context of a reference influenza virus data set^a^ and recent influenza viruses from the Netherlands

NA: neuraminidase; HA: haemagglutinin.

^a^ Reference dataset provided by the World Health Organization Collaborating Centre London through the European Centre for Disease prevention and Control for seasonal phylogenetic analysis. We gratefully acknowledge the authors, originating and submitting laboratories of the sequences retrieved from GISAID's Epiflu database that were used in this analysis (Supplement 2).

The HA and NA segments were highly similar to those of the 2018 A(H1N1)pdm09 and A(H3N2) influenza viruses in the Netherlands detected through the sentinel surveillance (Figure 2 and Figure 3).

HA: haemagglutinin.

^a^ Reference dataset provided by the World Health Organization Collaborating Centre London through the European Centre for Disease prevention and Control for seasonal phylogenetic analysis. We gratefully acknowledge the authors, originating and submitting laboratories of the sequences retrieved from GISAID's Epiflu database that were used in this analysis (Supplement 2).

The evolutionary history was inferred using the neighbour-joining method [12]. The optimal tree is shown. The percentage of replicate trees in which the associated taxa clustered together in the bootstrap test (1,000 replicates) is shown next to the branches [13]. The tree is drawn to scale, with branch lengths in the same units as those of the evolutionary distances used to infer the phylogenetic tree. The evolutionary distances were computed using the Jukes-Cantor method [14] and are in the units of the number of base substitutions per site. All ambiguous positions were removed for each sequence pair. Evolutionary analyses were conducted in MEGA7 [15]. Common amino acid substitutions (black: HA1, orange: HA2) compared with the root virus are shown at branches that characterise the cluster of viruses after that branch. HA of the influenza A(H1N2) virus (red dot) clustered with HA of recent 2018 Dutch clade 6B.1 A(H1N1)pdm09 influenza viruses characterised by common amino acid substitution V250A.

Phylogenetic analysis was carried out as described in the Figure 2 legend. Common amino acid substitutions compared with the root virus are shown at branches that characterise the cluster of viruses after that branch. The NA of the influenza A(H1N2) virus (red dot) clustered with NA of recent 2018 Dutch influenza A(H3N2) viruses characterised by common amino acid substitutions I176M and P386S. The NA of influenza A(H1N2) virus is derived from recent HA clade 3C.2a A(H3N2) influenza virus and has in addition the I30L amino acid substitution.

**Figure 4:**
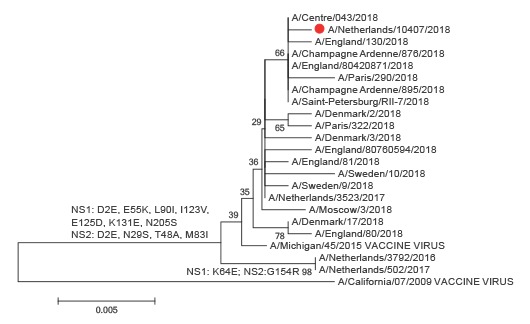
Phylogenetic analysis of the influenza A(H1N2) virus NS genome segment in the context of recent vaccine strains and selected seasonal influenza viruses representative of those circulating in the Netherlands in 2016 and 2017 and in other European countries in January and February 2018^a^

Because no full genomes of other 2018 Dutch influenza viruses were available, the other segments were compared with those from 100 A(H1N1)pdm09 and 200 A(H3N2) influenza viruses collected in 2018 from countries in the World Health Organization European Region. The NS segment was closely related to NS segments of 2018 A(H1N1)pdm09 viruses (Figure 4) and the PB2, PB1, PA, NP and M segments were closely related to the corresponding segments of 2018 A(H3N2) viruses (Supplement 1).

NS: non-structural protein.

^a^ Sequences were selected from ca 100 influenza A(H1N1)pdm09 viruses after phylogenetic analysis. We gratefully acknowledge the authors, originating and submitting laboratories of the sequences retrieved from GISAID's Epiflu database that were used in this analysis (Supplement 2).

Phylogenetic analysis was carried out as described in the Figure 2 legend. Red dot: influenza A(H1N2) virus described in this study.

We observed a number of nucleotide substitutions in the segments of this influenza A(H1N2) virus compared with the consensus sequences of other 2018 viruses. However, the majority of them were silent; unique amino acid substitutions were only identified in the M2 protein (V68I and D88N) and in the NA protein (I30L). M2 harboured S31N, similar to all recent influenza A(H3N2) viruses, causing resistance against the adamantane class of influenza antiviral drugs. In NA, no amino acid substitutions were detected that have been associated previously with reduced sensitivity to NA inhibitors, again similar to the vast majority of recent influenza A(H3N2) viruses. Furthermore, eight influenza A(H3N2) viruses isolated during the 2017/18 season in the sentinel surveillance in the Netherlands that had an NA protein sequence similar to that of the influenza A(H1N2) virus were tested for susceptibility to oseltamivir and zanamivir in the neuraminidase inhibition assay as described [4]. All eight were inhibited normally by oseltamivir (median IC_50_: 0.20; range: 0.17–0.28 nM) and zanamivir (median IC_50_: 0.31; range: 0.21–0.38 nM). Together, these findings indicate that this influenza A(H1N2) virus was highly likely to be sensitive to oseltamivir and zanamivir. As the HA protein sequence was similar to that of recent influenza A(H1N1)pdm09 viruses and interim 2017/18 season estimates of vaccine effectiveness for Europe showed 55–68% effectiveness in preventing laboratory-confirmed influenza A(H1N1)pdm09 virus infection [5], we consider that the currently used vaccine for the northern hemisphere offers protection against the influenza A(H1N2) virus. 

Unfortunately, attempts to isolate the A(H1N2) virus in cell culture and embryonated chicken eggs were unsuccessful, probably because of the low viral load in the clinical specimen at the time of sampling as shown by a Ct value of 29.2 in the RT-PCR targeting M. Experimental reassortant influenza virus with HA from A(H1N1)pdm09 influenza virus and the other segments from influenza A(H3N2) virus showed only slightly attenuated growth characteristics in human tracheobronchial epithelial (HTBE) cells with reduced amount of virus produced early in infection compared to parental A(H3N2) virus [6], suggesting that highly likely the current reassortant influenza A(H1N2) virus is capable of substantial growth. However, combined with the NS segment of influenza A(H1N1)pdm09 virus this effect might have an impact on the success of isolation of the current reassortant influenza A(H1N2) virus, especially when combined with low viral load in the clinical specimen. Further phenotypic characterisation of the virus awaits rescue of the virus using reverse genetics techniques.

## Epidemiological and virological follow-up

The patient consulted the GP again on 8 March with otitis media for which antibiotic treatment (amoxicillin 40 mg/kg 3 daily doses for 7 days) was prescribed. Within a few days, the patient made a full recovery. Contact investigation revealed that the parents had been ill in the last two weeks of February with symptoms of gastro-intestinal infection before onset of disease in the index case. No laboratory diagnostics was performed. None of the parents had close contacts with animals before the disease episode. The patient visited day care a couple of days per week; after onset of disease, the patient remained at home. In the day care centre, more than 20 children from different groups (babies 0–1-year-old, toddlers 1–ca 2.5-years-old and tots ca 2.5–4-years-old) were reported ill from mid-January through February. We do not know how many of these children had ILI or laboratory-confirmed influenza. However, given the fact that the influenza epidemic was at its peak at that time, it is likely that some of these children had influenza. From the end of February, children were reported ill only sporadically. In the sentinel surveillance and in the GP and hospital laboratory surveillance of influenza virus detections, no other influenza A(H1N2) cases were detected up to 20 March. The main diagnostic laboratory in the region was contacted to submit all type A influenza virus-positive specimens collected in March for subtyping; no reassortant viruses were identified among specimens from 24 patients; one of them was 5-weeks-old, while the rest had a median age of 73 years (range: 31–91 years).

## Discussion

Human seasonal reassortant A(H1N2) influenza viruses harbouring the H1 from human seasonal A(H1N1) and seven segments from human seasonal influenza A(H3N2) virus have been detected before during an outbreak in China in 1988/89 and from 2000 to 2003 starting in Asia and spreading worldwide [7-10]. However, the latter failed to co-circulate in the long term or to replace the A(H1N1) or A(H3N2) influenza viruses that were in circulation at the time. A sporadic case of seasonal reassortant A(H1N2) influenza virus harbouring the H1 from human influenza A(H1N1)pdm09 virus and the other seven segments from human seasonal influenza A(H3N2) virus was detected in India during the influenza pandemic in 2009 [11]. In a mixture of reassortants generated by in vitro coinfection with seasonal A(H1N1)pdm09 and A(H3N2) influenza viruses, sporadic virus with the current 2:6 constellation of genome segments was detected [6]. Furthermore, in a transmission experiment with guinea pigs infected with this mixture of reassortant viruses, the influenza A(H1N2) virus with the NS segment from influenza A(H1N1)pdm09 virus was not detected in contact animals [6]. 

In conclusion, this is the first time a seasonal reassortant A(H1N2) influenza virus with HA and NS from influenza A(H1N1)pdm09 virus and the other six segments of influenza A(H3N2) virus is reported in a human natural infection. The patient recovered fully and there is no evidence of extended spreading of the reassortant virus in humans. Published evidence indicates limited capacity for transmission of seasonal reassortant A(H1N2) influenza viruses [6,11]. However, laboratories, especially national influenza centres, should stay vigilant for reassortant human seasonal influenza viruses with mixed genome segments from A(H1N1)pdm09 and A(H3N2) influenza viruses.
